# Musculoskeletal Ultrasound and the Decision to Switch Therapy in Patients With Established Rheumatoid Arthritis

**DOI:** 10.7759/cureus.105718

**Published:** 2026-03-23

**Authors:** Vincent Tran, Thanda Aung, Erin Bauer, Holly Wilhalme, Chesca Barrios, Jordan Jacquez, Samantha Duong-Brett, Elizabeth Miller, Ernest Maningding, Benedict Tiong, Ruta Tesfamicael, Tracy Driver, Gihyun Myung, Chen Xie, Lisa Zhu, David Elashoff, Veena K Ranganath

**Affiliations:** 1 Rheumatology, University of California Los Angeles David Geffen School of Medicine, Los Angeles, USA; 2 Rheumatology, Virginia Mason Medical Center, Seattle, USA; 3 Statistics, University of California Los Angeles David Geffen School of Medicine, Los Angeles, USA; 4 Rheumatology, Park Nicollet Clinic and Specialty Center, St. Louis Park, USA; 5 Rheumatology, Alameda Health System, Oakland, USA; 6 Rheumatology, Catalina Pointe Arthritis and Rheumatology Clinic, Tucson, USA; 7 Rheumatology, Optum Health, Anaheim, USA; 8 Rheumatology, Seoul National University, Seoul, KOR

**Keywords:** disease modifying agent, flare up, musculoskeletal ultrasound, rheumatoid arthritis, switching therapy

## Abstract

Introduction

Musculoskeletal ultrasound (MSUS) with power Doppler ultrasound (PDUS) or gray-scale ultrasound (GSUS) has been increasingly used to detect active synovitis. This study evaluated PDUS/GSUS scores and clinical disease activity scores in patients with established rheumatoid arthritis (RA) whose rheumatologist was considering a therapy switch.

Methods

Thirty-two patients with RA were enrolled. Clinical Disease Activity Index (CDAI), Disease Activity Score-28 (DAS28), and PDUS/GSUS scores were obtained. Pearson correlation coefficients were used to evaluate the relationship of PDUS-24 and GSUS-24 with clinical composite measures. Demographics, disease activity measures, MSUS scores, and patient-reported outcomes were examined between switchers and non-switchers.

Results

Of the 32 patients enrolled, 59% (n=19) switched therapy. Switchers had numerically higher DAS28, CDAI, CRP/ESR, and PDUS/GSUS scores, but these metrics were not statistically significantly different from those of non-switchers. Among patients with concordant findings, 80% (n=8/10) of those with DAS28 ≥3.2 and high PDUS (>5) switched therapy, whereas only 25% (n=1/4) of patients with DAS28 <3.2 and low PDUS (≤5) did so. In the discordant group, therapy was switched in 60% of patients with high DAS28/low PDUS (n=6/10) and 60% of those with low DAS28/high PDUS (n=3/5). Physician global assessment and physician-reported RA flare were higher among therapy switchers (t(29) = -2.96, p=0.006; and Fisher’s exact p=0.03, respectively). Non-switchers were more satisfied with their treatment compared to switchers (8.8 vs 5.3, t(30) = 4.42, p<0.001).

Conclusion

Among patients with established RA, disease-modifying antirheumatic drug (DMARD) switchers had numerically higher CDAI, DAS28, and PDUS/GSUS scores compared with non-switchers. Eighty percent of patients with both increased DAS28 and PDUS switched therapy, compared with 60% of those with discordant findings, including high DAS28/low PDUS and vice versa. The PDUS/DAS28-discordant population faces inherently difficult therapeutic decisions and highlights the lack of guidance for physicians and patients.

## Introduction

The therapeutic landscape for rheumatoid arthritis (RA) has been revolutionized by the early introduction of disease-modifying antirheumatic drugs (DMARDs), leading to substantial improvements in clinical outcomes [1-3]. Current American College of Rheumatology (ACR) and European Alliance of Associations for Rheumatology (EULAR) guidelines advocate treat-to-target strategies using validated clinical composite measures [4, 5]. However, traditional disease activity scores such as the Clinical Disease Activity Index (CDAI) and Disease Activity Score-28 (DAS28) have limitations and may not fully capture a patient’s true disease state [6, 7].

Two clinical scenarios highlight the disconnect between clinical assessment and actual disease activity. First, patients achieving clinical remission may continue to experience progressive joint damage, suggesting persistent subclinical inflammation [8, 9]. Second, patients with comorbidities such as fibromyalgia, obesity, and osteoarthritis frequently experience chronic pain unrelated to inflammatory activity, making it challenging to distinguish between chronic pain and active RA when relying solely on these validated clinical composite disease activity scores [10-12].

Musculoskeletal ultrasound (MSUS) has gained recognition as a potentially more accurate method for assessing RA disease activity through the detection of synovitis via power Doppler ultrasound (PDUS) and gray-scale synovial hypertrophy (GSUS) [13, 14]. While landmark studies including the Targeting Synovitis in Early Rheumatoid Arthritis (TaSER) and ARCTIC trials examined MSUS in early RA cohorts, limited research has focused on patients with established RA in real-world clinical settings where rheumatologists are actively considering therapeutic modifications [15, 16].

The challenge of accurately assessing disease activity becomes more complex in established RA, where symptoms may arise from chronic joint damage or functional disability rather than active inflammation [17]. This complexity underscores the potential value of MSUS in distinguishing between inflammatory and non-inflammatory sources of patient symptoms.

This study evaluated the role of MSUS in therapeutic decision-making among patients with established RA whose rheumatologists were considering therapy optimization, characterizing the relationship between MSUS findings and clinical disease activity measures while examining factors influencing therapy modification decisions.

## Materials and methods

Patient population

This prospective study enrolled patients with established RA from outpatient rheumatology clinics when their treating rheumatologists were actively considering therapy switching. The study received institutional review board approval (IRB#16-000342-AM-00026) and adhered to the principles of the Declaration of Helsinki, with all participants providing written informed consent. Patients were enrolled between January 19, 2017, and December 19, 2022, at the University of California, Los Angeles (UCLA) rheumatology clinics.

Inclusion criteria were fulfillment of the ACR 2010 Classification Criteria for RA, disease duration ≥2 years, age ≥18 years, CDAI ≥3, and active consideration by the primary rheumatologist for RA therapy modification. Exclusion criteria included pregnancy or planned pregnancy, active or untreated malignancies, and active infections.

Clinical assessment and patient-reported outcomes

Comprehensive clinical evaluation included calculation of CDAI and DAS28-ESR using standard methodology. Patient-reported outcome measures encompassed the Health Assessment Questionnaire Disability Index (HAQ-DI) [18], Routine Assessment of Patient Index Data 3 (RAPID3) [19], the American English FLARE-RA questionnaire [20, 21], and the RA Flare Questionnaire (RA-FQ) [22].

Visual analogue scales (VAS) assessed stiffness, fatigue, patient global assessment, and pain intensity. RA treatment satisfaction was evaluated using a 5-point Likert scale. Laboratory parameters included ESR and CRP.

Musculoskeletal ultrasound protocol

MSUS examinations were performed using a GE LOGIQ E9 ultrasound system equipped with a 6-15 MHz linear probe. Standardized settings included red-yellow color mapping, Doppler frequency of 10.0 MHz, pulse repetition frequency of 0.8 kHz, and gain adjusted to just below the noise threshold.

A comprehensive 24-joint scanning protocol encompassed bilateral assessment of the wrists (midline wrist and radioulnar joints), metacarpophalangeal joints (MCP 1-5), proximal interphalangeal joints (PIP 2-5), and interphalangeal joints. Each joint was evaluated from multiple standardized views, generating 108 total images per patient. MSUS assessors remained blinded to all patient clinical data and outcomes (Table 1).

**Table 1:** Description of the MSUS scanning protocol for bilateral hands and wrists. MCPs: Metacarpophalangeal joints; PIPs: Proximal interphalangeal joints; MSUS: Musculoskeletal ultrasound.

Joint	View	Power Doppler synovitis	B-mode synovial hypertrophy (gray scale)	Total images obtained
Bilateral wrists, position: 0 degrees	Dorsal longitudinal midline (radiocarpal, intercarpal)	(2 images)	(2 images)	4
	Dorsal long/short radioulnar	(4 images)	(4 images)	8
Bilateral MCPs (1-5), position: 0 degrees	Dorsal long/short	(20 images)	(20 images)	40
	Volar longitudinal (except MCP1)	(8 images)	(8 images)	16
Bilateral PIPs (2-5) and IP	Dorsal/volar longitudinal	(20 images)	(20 images)	40
Total				108

Each image underwent semi-quantitative scoring using the validated LAJAX scoring system, with power Doppler and gray-scale findings scored from 0 to 3 [16]. For each joint, the maximum score across all views was recorded, and composite PDUS-24 and GSUS-24 scores were calculated by summing individual joint scores (possible range: 0-72). Primary referring rheumatologists remained blinded to all MSUS findings to avoid influencing clinical decision-making.

Inter- and intra-reader reliability were assessed by two rheumatology fellows who independently scored and re-scored two complete patient scans (154 images each). Intra-reader reliability demonstrated excellent agreement for PDUS (ICC 0.91-0.913, κ 0.84-0.86) and good agreement for GSUS (ICC 0.75-0.8, κ 0.7-0.72). Inter-reader reliability was excellent for PDUS (ICC 0.93-0.94, κ 0.84-0.86) and good for GSUS (ICC 0.77, κ 0.65-0.7).

Statistical analysis

Our initial power calculation suggested that a sample size of 40 would provide 80% power to detect correlations of at least 0.41 between PDUS and other study measures, assuming a two-sided significance level of 0.05.

Descriptive statistics were calculated for all study measures. Disease activity measures were compared between RA therapy switchers and non-switchers using independent t-tests for continuous variables and chi-square tests or Fisher’s exact tests for categorical variables. Pearson correlation coefficients were used to evaluate relationships between PDUS-24, GSUS-24, and other study measures. Statistical significance was set at p<0.05.

## Results

Patient characteristics

Thirty-five patients with RA were initially enrolled, with three lost to follow-up, yielding a final cohort of 32 patients. The cohort was predominantly female (93.8%, n=30) and seropositive (90.6%, n=29), with a mean age of 56 years (SD 15.0) and a mean disease duration of 9.8 years (SD 8.0). Prior exposure to a conventional synthetic DMARD (csDMARD) was present in nearly all patients (96.9%, n=31).

Nineteen patients (59%) ultimately switched therapy, while 13 (41%) did not. Therapy switchers were significantly younger than non-switchers (51.1 vs 63.2 years, t(30) = 2.40; p=0.02). Non-switchers had a higher frequency of prior bDMARD use compared to switchers, though this difference was not significant (92.3%, n=12 vs 68.4%, n=13; Fisher’s exact test p=0.19). Documented reasons for not switching therapy included stable or improved CDAI (n=4), current medication dose optimization (n=2), patient preference against change (n=5), and the determination that symptoms were non-inflammatory in nature (n=2) (Table 2). 

**Table 2:** Comparison of demographics between DMARD non-switchers and switchers. Percentages are calculated based on the number of non-missing observations for each variable; therefore, denominators may vary across variables. ACPA: Anti-citrullinated protein antibodies; RF: Rheumatoid factor; DMARDs: Disease-modifying antirheumatic drugs; csDMARDs: Conventional synthetic DMARDs; bDMARDs: Biologic DMARDs; tsDMARDs: Targeted synthetic DMARDs.

Switched DMARD(s)	All Participants (n=32)	No (n=13)	Yes (n=19)	P-value
Demographics				
Age (years), mean (SD)	56.0 (15.0)	63.2 (11.8)	51.1 (15.2)	0.02
Female, n (%)	30 (93.8%)	11 (84.6%)	19 (100%)	0.16
Disease duration (years), mean (SD)	9.8 (8.0)	12.9 (9.8)	8.0 (6.5)	0.12
Seropositive, n (%)	29 (90%)	9 (69.2%)	18 (94.7%)	0.05
ACPA positive, n (%)	24 (75.0%)	9 (69.2%)	15 (78.9%)	0.62
RF positive, n (%)	19 (59.4%)	7 (53.8%)	12 (63.2%)	0.72
Never smoker, n (%)	26 (81.2%)	11 (84.6%)	15 (78.9%)	1
Patient-reported comorbidities, mean (SD)	4.3 (3.1)	5.5 (3.6)	3.5 (2.6)	0.07
Charlson Comorbidity Index, mean (SD)	0.2 (0.6)	0.5 (0.9)	0.0 (0.0)	0.01
Radiographic measures				
Erosions, n (%)	16 (50.0%)	7 (53.8%)	9 (47.3%)	1
Joint space narrowing, n (%)	21 (65.6%)	9 (69.2%)	12 (63.2%)	0.67
Osteoarthritis, n (%)	16 (50.0%)	8 (61.5%)	8 (42.1%)	0.95
Race, n (%)				0.71
Non-Hispanic White	12 (37.5%)	4 (30.8%)	8 (42.1%)	
Other	20 (62.5%)	9 (69.2%)	11 (57.9%)	
Prior DMARDs				
csDMARDs, n (%)	31 (96.9%)	12 (92.3%)	19 (100.0%)	0.41
bDMARDs, n (%)	25 (78.1%)	12 (92.3%)	13 (68.4%)	0.19
tsDMARDs, n (%)	4 (12.5%)	1 (7.7%)	3 (15.8%)	0.62

Concordant and discordant disease activity patterns

The most clinically relevant finding emerged from the analysis of concordant versus discordant disease activity patterns. Among 10 patients with both high DAS28 (≥3.2) and high PDUS (>5), representing concordant high disease activity, 80% (n=8) switched therapy. Conversely, among 4 patients with both low DAS28 (<3.2) and low PDUS (≤5), representing concordant low disease activity, only 25% (n=1) switched therapy.

The discordant groups presented complex decision-making patterns. Among 10 patients with high DAS28 but low PDUS, 60% (n=6) switched therapy. Similarly, among 5 patients with low DAS28 but high PDUS, 60% (n=3) also switched therapy. This finding demonstrates that therapeutic decision-making becomes less predictable when clinical and ultrasound assessments provide conflicting information about disease activity.

Clinical disease activity and ultrasound findings

Table 3 examines MSUS scores, disease activity measures, and patient-reported outcomes in patients with RA who switched therapy and those who did not. While PDUS-24 and GSUS-24 scores were not significantly different between switchers and non-switchers, therapy switchers consistently demonstrated numerically higher ultrasound scores. Among the clinical disease activity measures, physician global assessment and physician-reported flare frequency were significantly higher in switchers than in non-switchers (t(29) = -2.96, p=0.006; and Fisher’s exact p=0.03, respectively).

**Table 3:** Comparison of MSUS scores, disease activity measures, and patient-reported outcomes between DMARD non-switchers and switchers Percentages are calculated based on the number of non-missing observations for each variable; therefore, denominators may vary across variables. PDUS: Power Doppler ultrasound; GSUS: Gray-scale ultrasound; DAS: Disease Activity Score; CDAI: Clinical Disease Activity Index; TJC: Tender joint count; SJC: Swollen joint count; VAS: Visual analogue scale; HAQ-DI: Health Assessment Questionnaire Disability Index; RAPID3: Routine Assessment of Patient Index Data 3; AmE FLARE-RA questionnaire: American English FLARE-RA questionnaire; RA-FQ: RA Flare Questionnaire.

Switched DMARD(s)	All participants (n=32)	No (n=13)	Yes (n=19)	P-value
Ultrasound measures				
PDUS-24, mean (SD)	11.9 (10.6)	7.0 (6.1)	12.3 (11.5)	0.16
PDUS >1, n (%)	27 (87.1%)	10 (83.3%)	17 (89.5%)	0.63
GSUS >1, n (%)	31 (100%)	12 (100%)	19 (100%)	NA
GSUS-24, mean (SD)	19.6 (11.6)	15.3 (8.4)	19.4 (12.0)	0.31
RA clinical disease activity				
DAS28-ESR (4-item), mean (SD)	4.0 (1.5)	3.5 (1.0)	4.2 (1.7)	0.2
DAS28 >3.2, n (%)	20 (68.9%)	6 (54.5%)	14 (77.8%)	0.24
CDAI, mean (SD)	15.9 (12.1)	12.0 (10.2)	18.9 (12.8)	0.12
CDAI >10, n (%)	18 (60.0%)	6 (46.2%)	12 (70.6%)	0.26
TJC28, mean (SD)	5.1 (5.3)	4.8 (6.3)	5.3 (4.7)	0.81
SJC28, mean (SD)	3.4 (4.6)	1.8 (2.3)	4.5 (5.4)	0.1
Physician global VAS, mean (SD)	3.6 (2.2)	2.4 (2.2)	4.4 (1.9)	0.006
Physician global >1, n (%)	25 (80.6%)	7 (53.8%)	18 (100%)	0.002
Patient global VAS, mean (SD)	4.0 (2.6)	3.0 (2.9)	4.7 (2.2)	0.07
Patient global >1, n (%)	26 (83.9%)	9 (69.2%)	17 (94.4%)	0.13
ESR, mean (SD)	26.5 (24.3)	20.4 (17.0)	30.6 (27.9)	0.27
CRP, mean (SD)	2.2 (4.0)	1.8 (3.2)	2.5 (4.6)	0.64
Physician flare, n (%)	7 (22.6%)	0 (0%)	7 (38.9%)	0.03
Patient flare, n (%)	12 (37.5%)	2 (15.4%)	10 (52.6%)	0.06
Patient-reported outcomes				
HAQ-DI, mean (SD)	0.9 (0.6)	0.7 (0.6)	1.0 (0.6)	0.19
RAPID3, mean (SD)	10.0 (6.3)	7.8 (6.7)	11.4 (5.8)	0.13
Satisfied with RA treatment, mean (SD)	6.8 (2.8)	8.8 (1.3)	5.3 (2.7)	<0.001
Current remission, n (%)	14 (45.2%)	5 (41.7%)	9 (47.4%)	1
Flare in last 3 months, n (%)				0.42
Yes, one	10 (31.2%)	5 (38.5%)	5 (26.3%)	
Yes, several times	17 (53.1%)	5 (38.5%)	12 (63.2%)	
AmE FLARE-RA questionnaire, mean (SD)	5.0 (3.0)	3.7 (3.3)	5.7 (2.6)	0.09
RA-FQ, mean (SD)	17.5 (12.7)	13.5 (13.7)	21.0 (11.2)	0.15
Pain VAS, mean (SD)	4.3 (2.8)	3.6 (3.2)	4.9 (2.6)	0.21
Morning stiffness VAS, mean (SD)	3.0 (2.4)	1.8 (2.2)	3.8 (2.2)	0.02
Fatigue VAS, mean (SD)	4.5 (2.7)	3.2 (3.0)	5.4 (2.0)	0.02

Additional clinical parameters, including DAS28, CDAI, tender joint count, swollen joint count, patient global assessment, ESR, and CRP, were all numerically higher in the switcher group, although statistical significance was not achieved, likely due to the limited sample size.

Patient-reported outcomes

Patient-reported outcomes revealed significant differences between groups. Therapy switchers were significantly younger than non-switchers (51.1 vs 63.2 years, t(30) = 2.40; p=0.02). Non-switchers reported significantly lower morning stiffness VAS (t(29) = -2.39, p=0.024), fatigue VAS (t(29) = -2.48, p=0.02), and work productivity impairment (t(27) = -2.58, p=0.02). Most notably, therapy switchers expressed significantly lower satisfaction with their current RA treatment regimen (5.3 vs 8.5, t(30) = 4.42, p<0.001).

Correlations between ultrasound and clinical measures

Table 4 describes the correlations of PDUS-24 and GSUS-24 with comorbidities, disease activity measures, and patient-reported outcomes. PDUS-24 and GSUS-24 demonstrated excellent correlation with each other (r=0.94, p<0.001), confirming the complementary nature of these ultrasound modalities. PDUS-24 also correlated significantly with DAS28 (r=0.43, p=0.01), CDAI (r=0.41, p=0.02), and HAQ-DI (r=0.39, p=0.02), supporting the construct validity of ultrasound assessment. The correlation between PDUS-24 and DAS28 was notably stronger among switchers than among non-switchers (ρ=0.48 vs ρ=0.16).

**Table 4:** Correlations between ultrasound measures, comorbidities, and disease activity indices. PDUS-24: Power Doppler Ultrasound Score-24; GSUS-24: Grey Scale Ultrasound Score-24; DAS28/ESR: Disease Activity Score in 28 joints using Erythrocyte Sedimentation Rate; CDAI: Clinical Disease Activity Index; HAQ-DI: Health Assessment Questionnaire Disability Index; RAPID3: Routine Assessment of Patient Index Data 3.

Variable	PDUS-24	P-value	GSUS-24	P-value
PDUS-24	-	-	0.94	<0.0001
GSUS-24	0.94	<0.0001	-	-
Charlson Comorbidity Index	0.09	0.66	0.08	0.66
Elixhauser Index	-0.09	0.51	-0.19	0.23
Patient-Reported Comorbidities	0.04	0.93	-0.06	0.74
DAS28/ESR	0.43	0.02	0.34	0.05
CDAI	0.41	0.02	0.3	0.1
HAQ-DI	0.39	0.02	0.28	0.1
RAPID3	0.28	0.1	0.22	0.21

## Discussion

This study examined the role of MSUS in therapeutic decision-making among patients with established RA whose rheumatologists were actively considering therapy modification. Our findings reveal that while RA therapy switchers demonstrated higher disease activity across multiple measures, the decision to change treatment was most complex when clinical and ultrasound assessments provided conflicting information.

Clinical implications of disease activity concordance

The finding that 80% of patients with concordant high clinical and ultrasound disease activity switched therapy, while only 25% with concordant low activity switched, supports the clinical utility of combined assessment approaches. When both clinical composite measures and ultrasound findings align to indicate either high or low disease activity, therapeutic decisions become more straightforward and predictable.

This pattern contrasts with early RA studies in which landmark trials such as ARCTIC and TaSER did not demonstrate clear superiority of ultrasound-guided treatment over conventional clinical assessment alone [15, 16]. Established RA presents unique diagnostic challenges due to accumulated joint damage, functional disability, and complex pain patterns that may not directly correlate with active inflammation [17, 23, 24]. In this context, MSUS may provide unique value by distinguishing between symptoms arising from chronic damage and those due to ongoing inflammatory activity.

Supporting this concept, a recent study of poly-refractory established RA patients found that 40% had no evidence of inflammation on MSUS, suggesting a non-inflammatory phenotype that had potentially been cycled unnecessarily through multiple therapies [19].

The challenge of discordant disease activity

The most clinically relevant finding is the therapeutic uncertainty created by discordant clinical and ultrasound assessments. The observation that 60% of patients in both discordant groups switched therapy highlights the complexity of decision-making when these modalities provide conflicting information.

Clinical Examples of Low Clinical/High MSUS Activity

Evidence from multiple studies supports the existence of RA patients in clinical remission who have detectable subclinical inflammation on MSUS or MRI. This scenario represents well-documented subclinical inflammation in the RA literature. Studies have demonstrated persistent GSUS and PDUS findings among patients achieving clinical remission by DAS28 or ACR criteria [25, 26]. Even stringent composite remission definitions do not eliminate ultrasound-detected synovitis in some patients with RA [27]. The clinical significance is substantial, as PDUS positivity in clinically quiescent patients predicts structural progression, relapse following DMARD tapering, and functional impairment [28-30]. In addition, intensifying therapy in patients with subclinical synovitis may reduce disease activity and prevent relapse [31].

Clinical Examples of High Clinical/Low MSUS Activity, i.e., Non-Inflammatory Pain

This pattern reflects the complex nature of pain in patients with RA. Non-inflammatory pain sources, including fibromyalgia, obesity, and psychosocial factors, can significantly elevate composite disease activity scores without reflecting actual inflammatory burden [10-12, 32, 33]. Fibromyalgia, present in more than 20% of patients with RA, can drive higher patient global assessments and tender joint counts independent of synovial inflammation [8, 9]. Importantly, PDUS and GSUS scores appear to remain unaffected by the presence of fibromyalgia, potentially offering a more accurate reflection of inflammatory activity [34].

Physician and patient factors in decision-making

The finding that physician global assessment and physician-reported flares were significantly higher in therapy switchers underscores the continued importance of clinical judgment in therapeutic decision-making. Despite the availability of standardized measures and objective imaging, physician assessment remains critical in treatment modification decisions.

The significantly lower treatment satisfaction among switchers (5.3 vs 8.5, p<0.001) likely reflects both patient symptoms and physician communication about treatment inadequacy, emphasizing the importance of shared decision-making approaches that incorporate both objective measures and patient preferences.

Study limitations

Several important limitations should be acknowledged. The restriction of MSUS examination to the hands and wrists may have missed disease activity in other joints, though limited joint assessments have shown good correlation with extended examinations [35]. The relatively small sample size (n=32) likely contributed to the lack of statistical significance for several numerically different measures between groups and limits the generalizability of the findings. Even with the reduced sample size, we were still able to detect meaningful correlations, although the study was originally powered to detect correlations of 0.41. This suggests that the reduced sample size remained sufficient for the primary analyses, although statistical power may have been lower than originally planned. In addition, recruitment was challenging during the COVID-19 pandemic, which contributed to the smaller sample size.

The single-center design with a predominantly female (93.8%) and seropositive (90.6%) cohort may not reflect the broader RA population in diverse clinical settings. The cross-sectional design captures only a single time point in therapeutic decision-making, preventing assessment of whether switching decisions ultimately improved clinical outcomes. Longitudinal follow-up studies would provide valuable insight into the consequences of switching versus non-switching decisions.

Selection bias is inherent, as patients were enrolled when rheumatologists were already considering therapy modification, which may not reflect routine clinical decision-making. The study did not standardize the reasons for considering therapy changes or assess important patient-centered factors such as medication preferences, cost considerations, or psychosocial factors that influence real-world therapeutic decisions.

Finally, while blinding rheumatologists to ultrasound findings was methodologically appropriate, it limits clinical applicability, since contemporary practice increasingly integrates point-of-care ultrasound into decision-making. Future studies should examine therapeutic decisions when ultrasound information is available to treating physicians.

## Conclusions

Among patients with established RA being considered for therapy modification, those who ultimately switched treatment demonstrated consistently higher disease activity across clinical, ultrasound, and patient-reported measures. Therapeutic decisions were most predictable when clinical composite measures and ultrasound findings were concordant: 80% (n=8/10) of patients with both high DAS28 and high PDUS switched therapy, while only 25% (n=1/4) of those with both low scores did so. In contrast, 60% of patients with discordant findings switched therapy regardless of which measure was elevated, highlighting the complexity of decision-making in this population and the critical need for evidence-based guidance when clinical and imaging assessments provide conflicting information.
